# Genome Sequence of the Fish Brain Bacterium Clostridium tarantellae

**DOI:** 10.1128/MRA.01575-19

**Published:** 2020-04-16

**Authors:** Luca Bano, Matthias Kiel, Gabriele Sales, Andrew C. Doxey, Michael J. Mansfield, Haleluya T. Wami, Marco Schiavone, Ornella Rossetto, Marco Pirazzini, Ulrich Dobrindt, Cesare Montecucco

**Affiliations:** a Microbiology Laboratory, Istituto Zooprofiilattico Sperimentale delle Venezie, Villorba, Italy; b Institute of Hygiene, University of Muenster, Muenster, Germany; c Department of Biology, University of Padua, Padua, Italy; d Department of Biology, University of Waterloo, Waterloo, Canada; e Department of Biomedical Sciences, University of Padua, Padua, Italy; f CNR Institute of Neuroscience, Padua, Italy; Indiana University, Bloomington

## Abstract

Eubacterium tarantellae was originally cultivated from the brain of fish affected by twirling movements. Here, we present the draft genome sequence of *E. tarantellae* DSM 3997, which consists of 3,982,316 bp. Most protein-coding genes in this strain are similar to genes of *Clostridium* bacteria, supporting the renaming of *E. tarantellae* as Clostridium tarantellae.

## ANNOUNCEMENT

The brain is generally considered a bacterium-free organ that can be infected only after alteration of the blood-brain barrier. However, Udey et al. (1) reported the unique isolation of a bacterium from the brain of apparently healthy fish displaying a rapid turning-around movement (twirling). This behavioral change is most likely to be the consequence of an alteration of the brain circuits that control body movements. Accordingly, this bacterium was named Eubacterium tarantellae, described as a non-spore-forming anaerobe and suggested to be the first of a novel class of anaerobic fish pathogenic bacteria (2). No further characterization was reported, though it was isolated from fish larval gut flora (3) and recently associated with a human case of joint septic arthritis (4).

*E. tarantellae* DSM 3997 was cultivated in synthetic thioglycolate broth under anaerobiosis. DNA was automatically extracted (Microlab STARlet; Hamilton, Switzerland) using a MagMAX total nucleic acid isolation kit (Ambion/Life Technologies, USA). Whole-genome sequencing was performed using an Illumina MiSeq sequencing platform. To prepare 500-bp paired-end libraries of *E. tarantellae*, we used the Nextera XT DNA library preparation kit (Illumina, USA). Libraries were sequenced using V2 sequencing chemistry. Quality trimming of 2,896,670 raw reads (1,448,335 pairs) was performed using Sickle v1.33 (https://github.com/najoshi/sickle) with the options –l 30 and –q 28 and resulted in 2,213,220 single reads corresponding to 1,106,610 paired-end reads. *De novo* assembly of the processed reads was performed with SPAdes v3.10.1 using default parameters (5). The assembly resulted in 713 contigs, validated using QualiMap v2.1, with an *N*_50_ contig size of 11.440 bp. The draft genome consists of 3,979,886 bp with a 25.8% GC content and an average coverage of 149×. The genome assembly was estimated to be 98.5% complete using CheckM (6) with only 5% redundancy.

Automatic gene prediction was performed using PGAP v4.10 (www.ncbi.nlm.nih.gov/genome/annotation_prok/) (7). The draft genome encodes 3,069 predicted protein-coding genes, including 2,274 genes with a predicted function and 795 genes coding for hypothetical proteins. Additionally, 11 rRNA and 72 tRNA genes were predicted. Furthermore, one contig includes a clustered regularly interspaced short palindromic repeat (CRISPR)-associated (Cas) operon including putative genes homologous to Cas3, Cas5, Cas7, and Cas8b/Csh1 from other clostridia.

**FIG 1:**
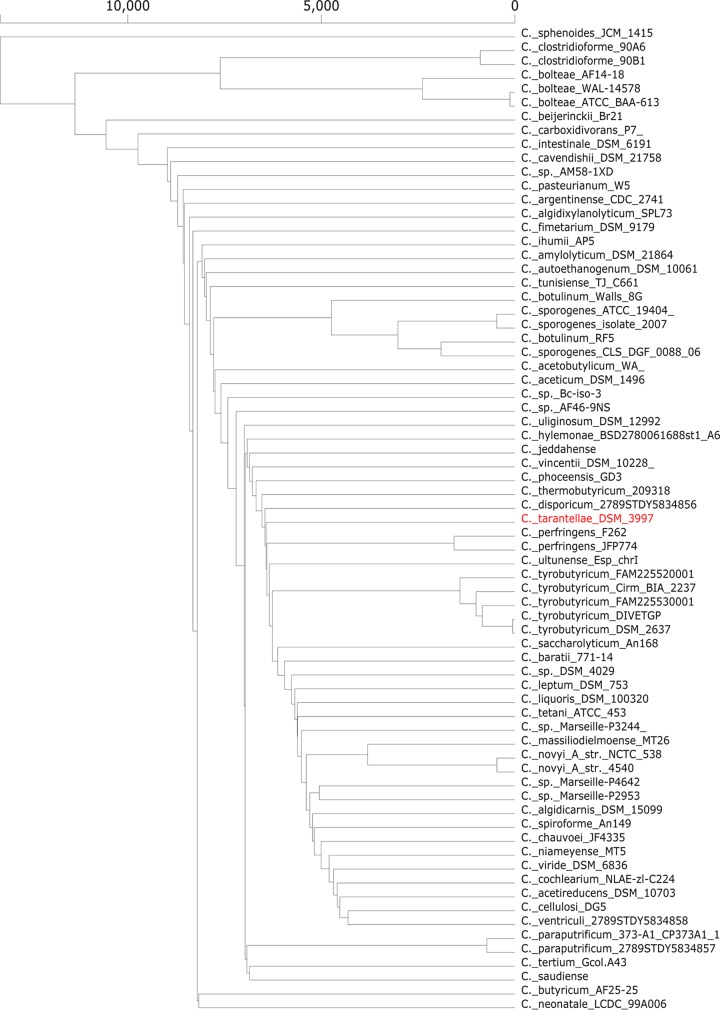
Comparison of the genome content of clostridial species based on the presence/absence of genes. The pan-genome presence/absence analysis of 71 *Clostridium* strains was performed using Roary v3.12.0 with default parameters. The generated tree was visualized using FriPan (https://github.com/drpowell/FriPan).

Phylogenetic analysis of taxonomic markers (8) showed that the *E. tarantellae* genome is closely related to that of Clostridium perfringens, followed by those of other *Clostridium* species. Pan-genome analysis based on the presence/absence of genes in publicly available clostridial genome sequences and in the *C. tarantellae* DSM 3997 genome was performed with Roary v3.12.0 (8) with default parameters and corroborated that C. perfringens and *C. tarantellae* are indeed closely related (Fig. 1). Due to the peculiar association between *E. tarantellae* and fish brain/movement alteration, we searched the genome for potential neurotoxins possibly responsible for inducing twirling using the virulence factor database (VFDB) (9). No genes related to clostridial neurotoxins were found. However, a gene encoding α-perfringolysin-like pore-forming toxin from C. perfringens not known to induce alteration of movements is present. In addition, several putative toxins similar to the Clostridium septicum epsilon toxin ETX were identified. Furthermore, three leukocidins/hemolysins similar to the beta-channel-forming toxin NetE of C. perfringens and some clostridial-type phospholipases were predicted to be encoded in the *E. tarantellae* genome. Small cysteine-rich proteins that encode possible antimicrobial peptides or peptide toxins were also identified (e.g., CEFHDAMA_03104 and CEFHDAMA_00788) and found to be conserved in other clostridia. In addition, though *E. tarantellae* was not found to sporulate (1, 2), the genes encoding proteins involved in the execution of the sporogenesis program are present. The high similarity of the present genome to those of clostridial species strongly supports the suggestion that Eubacterium tarantellae should be renamed Clostridium tarantellae (10).

### Data availability.

This whole-genome shotgun project has been deposited at DDBJ/ENA/GenBank under the accession number WHJC00000000. The version described in this paper is version WHJC01000000. The raw reads are available at the Sequence Read Archive under the accession number PRJNA587720.

## References

[1] Udey L, Young RE, Sallman B 1977 Isolation and characterization of an anaerobic bacterium, *Eubacterium tararantellus* sp.nov., associated with striped mullet (*Mugil cephalus*) mortality in Biscayne Bay, Florida. J Fish Res Bd Can 34:402–409. doi:10.1139/f77-064.

[2] Udey LR 1978 Anaerobic bacteria as possible disease agents in fish. Marine Fish Rev 40:10–12.

[3] Akayli T, Erkan M, Yardimci RE, Ozgur C, Urku C 2015 Interaction of gut flora and bacterial pathogens of cultured common dentex (*Dentex dentex*). Isr J Aquacult. http://hdl.handle.net/10524/49173.

[4] Cointe A, de Ponfilly GP, Munier AL, Bachir M, Benmansour H, Crémieux AC, Forien M, Frazier A, Krief E, Cambau E, Jacquier H 2019 Native joint septic arthritis due to *Clostridium tarantellae*. Anaerobe 56:46–48. doi:10.1016/j.anaerobe.2019.02.004. 30753899

[5] Bankevich A, Nurk S, Antipov D, Gurevich AA, Dvorkin M, Kulikov AS, Lesin VM, Nikolenko SI, Pham S, Prjibelski AD, Pyshkin AV, Sirotkin AV, Vyahhi N, Tesler G, Alekseyev MA, Pevzner PA 2012 SPAdes: a new genome assembly algorithm and its applications to single-cell sequencing. J Comput Biol 19:455–477. doi:10.1089/cmb.2012.0021. 22506599 PMC3342519

[6] Parks DH, Imelfort M, Skennerton CT, Hugenholtz P, Tyson GW 2015 CheckM: assessing the quality of microbial genomes recovered from isolates, single cells, and metagenomes. Genome Res 25:1043–1055. doi:10.1101/gr.186072.114. 25977477 PMC4484387

[7] Petrenko P, Lobb B, Kurtz DA, Neufeld JD, Doxey AC 2015 MetAnnotate: function-specific taxonomic profiling and comparison of metagenomes. BMC Biol 13:92. doi:10.1186/s12915-015-0195-4. 26541816 PMC4636000

[8] Page AJ, Cummins CA, Hunt M, Wong VK, Reuter S, Holden MTG, Fookes M, Falush D, Keane JA, Parkhill J 2015 Roary: rapid large-scale prokaryote pan genome analysis. Bioinformatics 31:3691–3693. doi:10.1093/bioinformatics/btv421. 26198102 PMC4817141

[9] Liu B, Zheng D, Jin Q, Chen L, Yang J 2019 VFDB 2019: a comparative pathogenomic platform with an interactive Web interface. Nucleic Acids Res 47:D687–D692. doi:10.1093/nar/gky1080. 30395255 PMC6324032

[10] Lawson PA, Rainey FA 2016 Proposal to restrict the genus *Clostridium Prazmowski* to *Clostridium butyricum* and related species. Int J Syst Evol Microbiol 66:1009–1016. doi:10.1099/ijsem.0.000824. 26643615

